# Probiotic Microbes Sustain Youthful Serum Testosterone Levels and Testicular Size in Aging Mice

**DOI:** 10.1371/journal.pone.0084877

**Published:** 2014-01-02

**Authors:** Theofilos Poutahidis, Alex Springer, Tatiana Levkovich, Peimin Qi, Bernard J. Varian, Jessica R. Lakritz, Yassin M. Ibrahim, Antonis Chatzigiagkos, Eric J. Alm, Susan E. Erdman

**Affiliations:** 1 Division of Comparative Medicine, Massachusetts Institute of Technology, Cambridge, Massachusetts, United States of America; 2 Laboratory of Pathology, Faculty of Veterinary Medicine, Aristotle University of Thessaloniki, Thessaloniki, Greece; 3 Biological Engineering, Massachusetts Institute of Technology, Cambridge, Massachusetts, United States of America; 4 Broad Institute of MIT and Harvard, Cambridge, Massachusetts, United States of America; University Hospital of Münster, Germany

## Abstract

The decline of circulating testosterone levels in aging men is associated with adverse health effects. During studies of probiotic bacteria and obesity, we discovered that male mice routinely consuming purified lactic acid bacteria originally isolated from human milk had larger testicles and increased serum testosterone levels compared to their age-matched controls. Further investigation using microscopy-assisted histomorphometry of testicular tissue showed that mice consuming *Lactobacillus reuteri* in their drinking water had significantly increased seminiferous tubule cross-sectional profiles and increased spermatogenesis and Leydig cell numbers per testis when compared with matched diet counterparts This showed that criteria of gonadal aging were reduced after routinely consuming a purified microbe such as *L. reuteri*. We tested whether these features typical of sustained reproductive fitness may be due to anti-inflammatory properties of *L. reuteri*, and found that testicular mass and other indicators typical of old age were similarly restored to youthful levels using systemic administration of antibodies blocking pro-inflammatory cytokine interleukin-17A. This indicated that uncontrolled host inflammatory responses contributed to the testicular atrophy phenotype in aged mice. Reduced circulating testosterone levels have been implicated in many adverse effects; dietary *L. reuteri* or other probiotic supplementation may provide a viable natural approach to prevention of male hypogonadism, absent the controversy and side-effects of traditional therapies, and yield practical options for management of disorders typically associated with normal aging. These novel findings suggest a potential high impact for microbe therapy in public health by imparting hormonal and gonad features of reproductive fitness typical of much younger healthy individuals.

## Introduction

The main cellular source of testosterone in male mammals is the Leydig cell, which resides in clusters within the testicular interstitium. Adult Leydig cell androgen production is regulated by luteinizing hormone-mediated signals, originating from the hypothalamus-pituitary gland axis [1]. In males, an inevitable reduction of circulating testosterone occurs with increasing age [2]–[4]. The mechanisms governing this phenomenon remain largely unknown. Studies in both humans and rodents, however, suggest that low testosterone is due to age-related lesions in testes rather than irregular luteinizing hormone metabolism [1], [5]–[7].

The reduction of testosterone has been implicated in many adverse effects of aging in men, including reduced spermatogenesis, libido and sexual function, increased body fat, decreased muscle and bone mass, low energy levels, fatigue, poor physical performance, depressed mood, and impaired cognitive dysfunction [8]–[11]. Whether low testosterone can be considered the cause of a defined clinical entity in elderly men or not has been a matter of debate [12]. Additionally, different names have been used to describe this condition (male menopause, andropause, partial androgen deficiency of the aging male and late-onset hypogonadism) and different criteria have been used to define it, hence the discrepancies of epidemiological studies during reporting on its prevalence [12]–[13]. Nonetheless, there is an unquestionable consensus in that age-related progressive morphological and functional alterations of Leydig cells lead to low testosterone levels that, in turn, reduce reproductive fitness and affect the quality of life of the aging male [1], [4]–[5], [9]–[10], [12]–[13].

Various dietary factors and diet-induced obesity have been shown to increase the risk for late onset male hypogonadism and low testosterone production in both humans and mice. Testosterone deficiency and metabolic diseases such as obesity appear to inter-digitate in complex cause-and-effect relationships [14]–[17].

Recently we have found that the dietary supplementation of aged mice with the probiotic bacterium *Lactobacillus reuteri* makes them appear to be younger than their matched untreated sibling mice, at least in part by inducing beneficial integumentary effects that manifest as luxuriant hair [18] and inhibition of diet-induced obesity [19]. Interestingly, both these effects were linked with a CD4+ regulatory-T cell and Il-10-associated systematic down-regulation of the pro-inflammatory cytokine Il-17 [18]–[19]. These results indicate that gut microbiota induce modulation of local gastrointestinal immunity resulting in systemic effects on the immune system which activate metabolic pathways that restore tissue homeostasis and overall health. Indeed, in another study we discovered that aged mice eating *L. reuteri* show accelerated healing of skin wounds, which depends upon the suppressive arm of the immune system and the up-regulation of the pituitary gland neuropeptide hormone oxytocin (manuscript submitted for publication). During all these studies we consistently observed that young and aged mice consuming purified *L. reuteri* organisms had particularly large testes and a dominant male behavior.

Our previous studies and observations, taken together, led us to hypothesize that dietary *L. reuteri* may act to prevent age- and obesity-related testicular atrophy in mice. The results of the present study confirmed this hypothesis. The testes of probiotic-fed aged mice were rescued from both seminiferous tubule atrophy and interstitial Leydig cell area reduction typical of the normal aging process. Preservation of testicular architecture despite advanced age or high-fat diet coincided with remarkably high levels of circulating testosterone. The beneficial effects of probiotic consumption were recapitulated by the depletion of the pro-inflammatory cytokine Il-17.

Given the aggravated controversy about the benefits and side-effect risks of testosterone replacement therapy [9]–[10], [13], [20], dietary *L. reuteri* may provide an alternative natural approach to the prevention of late-onset male hypogonadism.

## Results

### *Lactobacillus reuteri* Consumption Increases Testicular Weight of Mice Fed with Normal Diet

**Figure 1:**
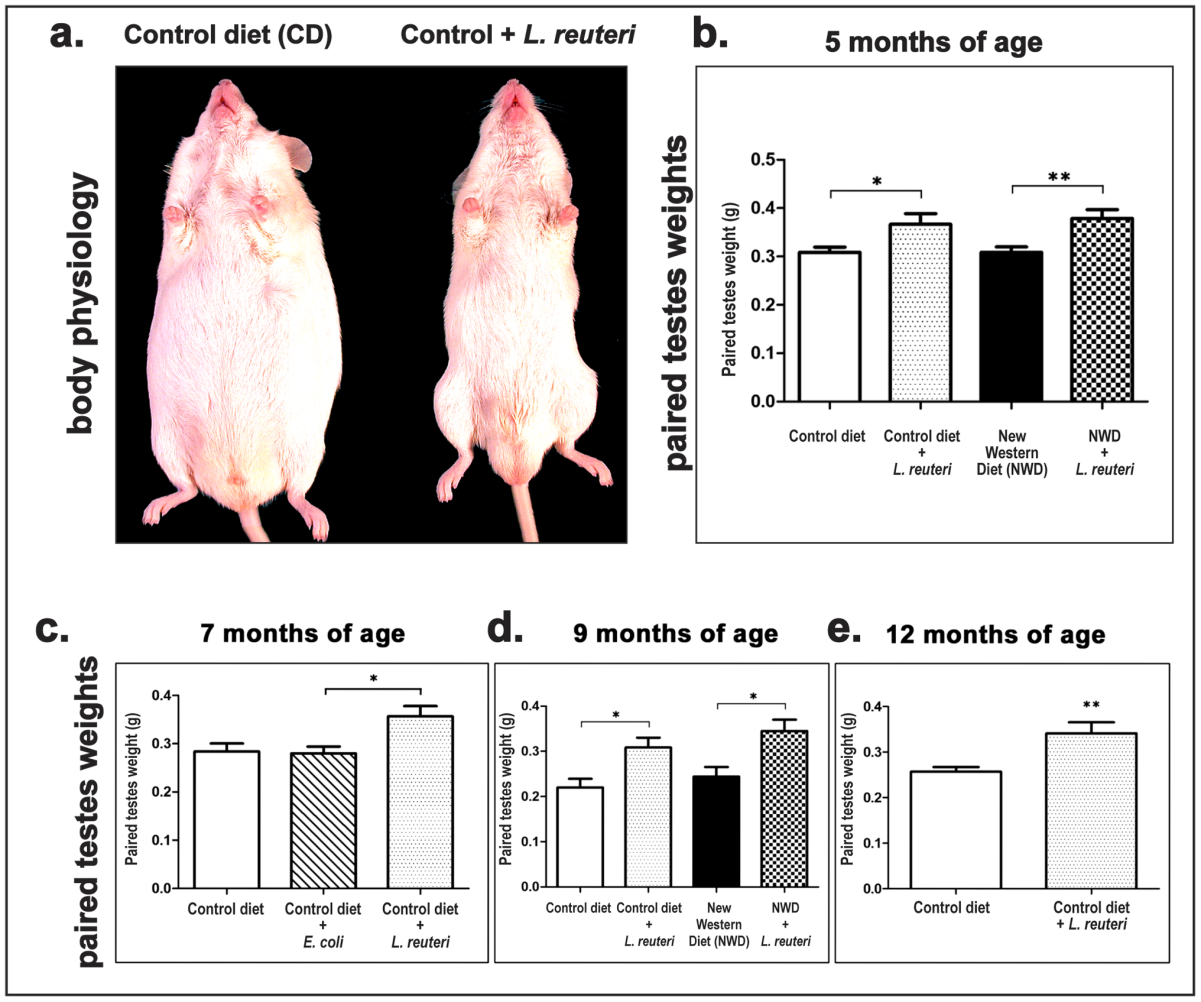
Consumption of probiotic microbes increases the weight of mouse testes. **a.** Gross appearance of outbred Swiss male mice with oral *Lactobacillus reuteri* treatment at 12 months of age. Testes of *L. reuteri*-fed mice are larger compared to control mice. **b–e.** Results of statistical analyses of testes weight at different time-points after starting treatment, including the (**b**) 5, (**c**) 7, (**d**) 9 and (**e**) 12 months of age time-points. *L. reuteri* consistently increased testicular weight in both control- and new western-diet-fed mice compared to age- and diet-matched controls. Dietary supplementation with *L. reuteri*, but not with *E coli* K12, increased testicular weight in control-diet-fed mice at age 7 months when compared to age- and diet-matched controls (**c**). Numbers on the y-axis of bar graphs correspond to the mean±SEM of testes weight or the testicular weight; *p<0.05, **p<0.001.

We observed unusually large testes and social dominance behaviors in male mouse models while feeding probiotic yogurt or purified lactic acid bacteria during obesity and cancer studies (Figure 1a). To test specific effects of probiotics on testes, outbred Swiss mice on normal (control) diet were given *Lactobacillus reuteri* ATCC 6475 using a dosage of 3.5×10^5^ organisms/mouse/day starting at 2 months of age and their testes were weighed at 3, 5, 7 and 10 months later. At each time-point the weight of testes from mice consuming probiotics was significantly higher than that of control mice. Specifically, at five months (CD vs CD+LR, *P* = 0.0408) (Fig. 1b), seven months (CD vs CD+LR, *P* = 0.0328) (Fig. 1c), nine months (CD vs CD+LR, *P* = 0.0178) (Fig. 1e), and 12 months of age (CD vs CD+LR, *P* = 0.0062) (Fig. 1e) the statistical significance of testes weight difference between probiotic-treated and non-treated groups of mice progressively increased with age. When the testes weight values were adjusted for body weight (testis weight/body weight ratio) the *L. reuteri* effect was more pronounced in all different age time-points examined (Fig. S1). While feeding of *L. reuteri* consistently increased the gonadal weights, consumption of a non-pathogenic strain of *Escherichia coli* (*E. coli*) K12 organisms did not affect testicular weight (CD+*E.coli* vs CD+LR, *P* = 0.026) (Fig. 1c) or the testicular/body weight ratio when compared at age 7 months, confirming the specificity of the probiotic organism effect (Fig. S1).

### *L. reuteri* Increases Testicular Weight of Mice on High-fat Diet

Since obesity has been reported to have an adverse effect on testicular function [14]–[17], we next sought to examine whether consumption of *L. reuteri* could increase testicular weight of obese mice consuming a commercially available New Westernized type of diet (NWD) with high fat and low fiber with substandard levels of B vitamins and Vitamin D mimicking human ‘fast food’ diets. We found that probiotic-fed mice had significantly increased testicular weight compared to age- and diet-matched controls at five months (NWD vs NWD+LR, *P* = 0.0096) (Fig. 1b) and nine months (NWD vs NWD+LR, *P* = 0.0491) of age (Fig. 1d). Similar to what we have recently reported, these mice with dietary *L. reuteri* supplements were rescued from diet-induced obesity and had normal body weight and lean physique [19]. Therefore, when testicular weight values adjusted for body weight (instead of absolute testes weight values) were analyzed, the significance of statistical differences was increased at both five month (NWD vs NWD+LR, *P* = 0.0007) and nine month time points (NWD vs NWD+LR, *P* = 0.0159) (Fig. S1).

### Testes from *L. reuteri-*treated Mice have Increased Seminiferous Tubule Cross-sectional Profile Areas

**Figure 2:**
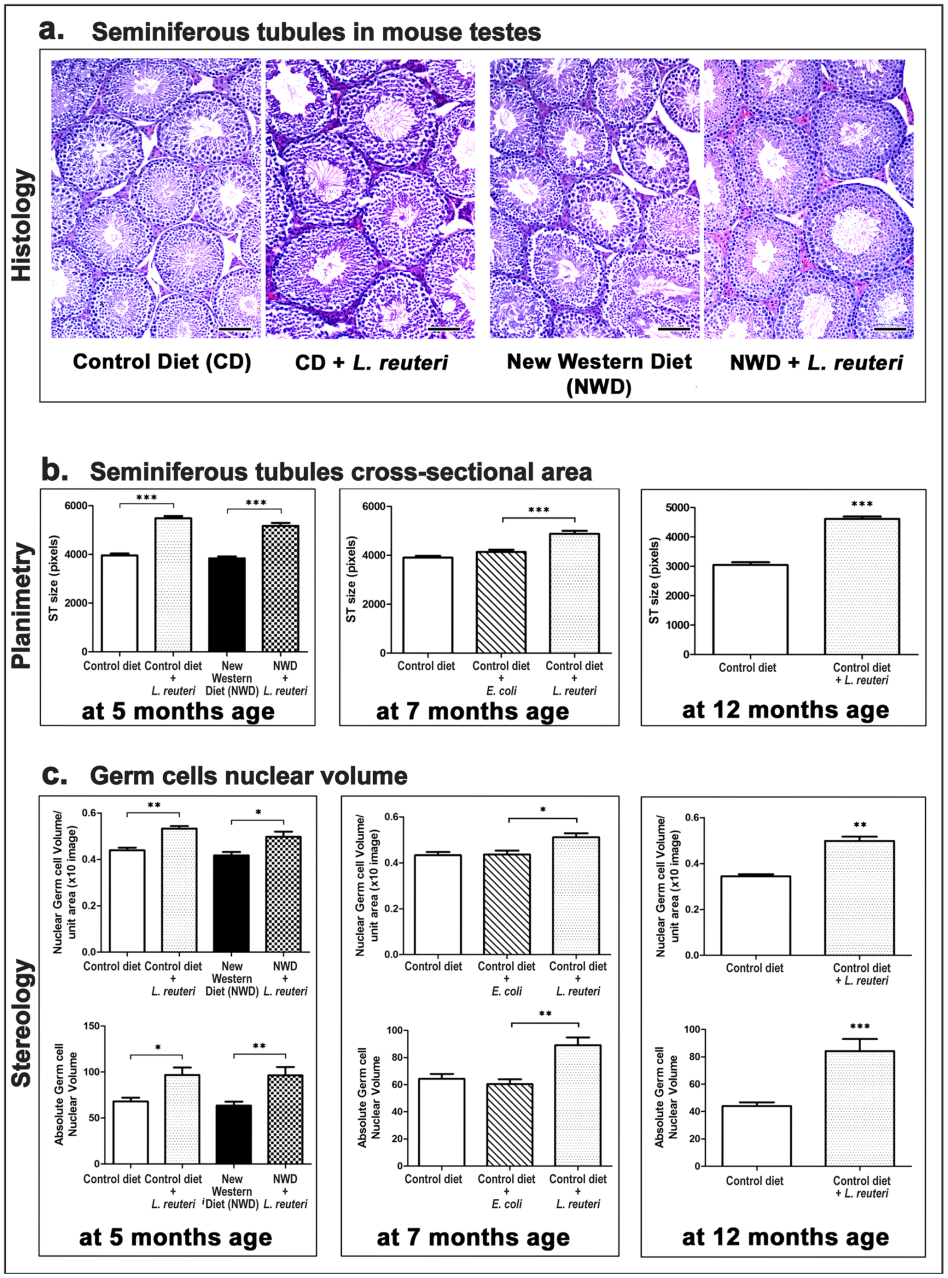
Dietary *L. reuteri* increases the cross-sectional profile area of seminiferous tubules and their germ cell population. **a.** Representative histology of seminiferous tubules cross-sectioned profiles of Swiss mice at the age of 5 months. Increased testicular weight due to *L. reuteri* consumption in both control- and New Western diet (NWD)-fed mice co-existed with a histologically discernible increase of seminiferous tubule (ST) cross-sectional area. Hematoxylin and eosin. Bars = 100 µm. **b.** Histomorphometrical assessment of circular ST cross-sectioned profiles size in different time points. The effect of *L. reuteri* remains highly significant with the progression of age. Numbers on the y axis of bar graphs correspond to the mean±SEM of ST area size; *** p<0.0001. **c.** Stereological morphometric assessment shows that *L. reuteri*-fed mouse testes have significantly increased nuclear volumes of germ cells. Numbers on the y axis of bar graphs correspond to the mean±SEM of “Nuclear volume of germ cells per unit area” or the “absolute nuclear volume of germ cells”; *p<0.05, **p<0.001, ***p<0.0001.

In order to explain the probiotic-associated increase in paired testicular mass, we applied microscopy–assisted histomorphometry and assessed critical parameters of testes architecture. We found that *L. reuteri* consumption did not affect the total number of seminiferous tubule (ST) profiles counted in histological sections of testes. At 5 months of age there was no difference in ST counts of both control and NWD-fed groups of mice. Similar results were obtained from the testes of mice at seven months and 12 months (data not shown). Despite the comparable numbers of ST profiles, we determined that testes from *L. reuteri-*treated mice had increased ST cross-sectioned profiles (Fig. 2a). To quantify this result we measured the area size of circular ST profiles in experimental groups. The statistical analysis of results confirmed our observations. At 5 months of age circular ST profiles from *L. reuteri*-fed mouse testes were significantly larger than those of their diet-matched counterparts (CD vs CD+LR, *P*<0.0001; NWD vs NWD+LR, *P*<0.0001). Similar pronounced statistical differences were found at seven months (CD vs CD+LR, *P<*0.0001; CD+*E.coli* vs CD+LR, *P*<0.0001) and 12 months of age (CD vs CD+LR, *P*<0.0001) (Fig. 2b). To determine whether increased cross-sectional area of ST profiles in *L. reuteri*-treated mice was due to increased germ cells, we further measured germ cell nuclear volumes using point-counting, stereology-based morphometry. We found that the nuclear volume of germ cells per unit size area (×10 image) was significantly higher in mice consuming *L. reuteri* at 5 months (CD vs CD+LR, *P = *0.0026; NWD vs NWD+LR, *P = *0.04), at 7 months (CD vs CD+LR, *P = *0.0103; CD+*E.coli* vs CD+LR, *P = *0.018) and at 12 months of age (CD vs CD+LR, *P = *0.0021) (Fig. 2c). Consequently, the absolute nuclear volume of germ cells per testis in those experimental groups was also significantly higher at 5 (CD vs CD+LR, *P = *0.0126; NWD vs NWD+LR, *P = *0.007), 7 (CD vs CD+LR, *P = *0.0026; CD+*E.coli* vs CD+LR, *P = *0.0026) and 12 months of age (CD vs CD+LR, *P = *0.0006) (Fig. 2c). When these results were expressed as the relative percentage of nuclear volume of germ cells per testis and analyzed, we found percentage values that were comparable between experimental groups (data not shown). This result suggests that the increase of testes weight due to *L. reuteri* consumption was proportional to the increase of the testicular germ cell population.

### Testes from *L. reuteri*-treated Mice have More Conspicuous Leydig Cell Areas

**Figure 3:**
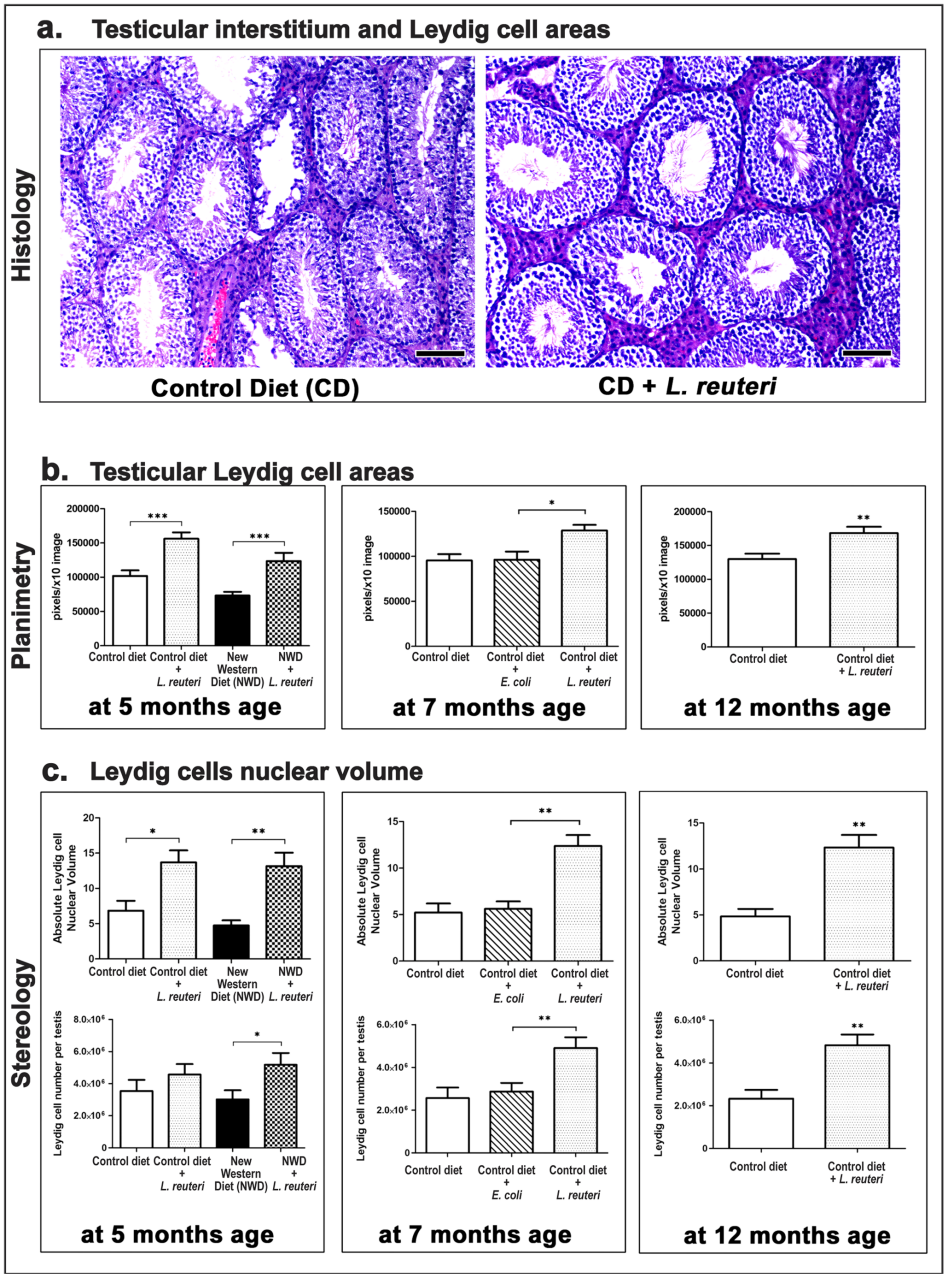
*L. reuteri* consumption increases the size of interstitial Leydig cell areas and Leydig cell numbers. **a.** Representative histology of Swiss mice testes at the age of 12 months. Leydig cell areas in the testicular interstitium of probiotic-fed mice are of increased size compared to the corresponding areas of control mice. Hematoxylin and eosin. Bars = 100 µm **b.** Histomorphometrical counts of Leydig cell areas at different time-points revealed the statistical significance of the *L. reuteri* effect. Numbers on the y-axis of bar graphs correspond to the mean±SEM of Leydig area size. **c.** Point-counting stereology counts reveal that *L. reuteri*-fed mouse testes have significantly increased nuclear volumes and an increased number of Leydig cells per testis. Numbers on the y axis of bar graphs correspond to the mean±SEM of “Nuclear volume of germ cells per unit area” or the “absolute nuclear volume of germ cells”; *p<0.05, **p<0.001, ***p<0.0001.

Our histopathological examination revealed that the *L. reuteri* effect on mouse testicular tissue was not limited to ST's but also extended to the interstitium. Specifically, the probiotic organism induced prominent Leydig cell accumulations in the interstitial tissue between the ST's (Fig. 3a). To quantify this observation further we morphometrically assessed Leydig cell areas in the different treatment groups. At 5 months of age there were significant differences in Leydig cell area counts of both control (CD vs CD+LR, *P<*0.0001) and NWD-fed groups of mice (NWD vs NWD+LR, *P* = 0.0002). The probiotic-associated increase of interstitial Leydig cell areas was sustained with advancing age at 7 (CD vs CD+LR, *P* = 0.0025; CD+*E.coli* vs CD+LR, *P* = 0.0251) and 12 months (CD vs CD+LR, *P* = 0.0012) (Fig. 3b).

Using the point-counting method, we found that the nuclear volumes of Leydig cells per unit size area (×10 image) was significantly higher in mice eating the probiotic organism at 5 months (CD vs CD+LR, *P = *0.0136; NWD vs NWD+LR, *P = *0.0055), at 7 months (CD vs CD+LR, *P = *0.0031; CD+*E.coli* vs CD+LR, *P = *0.0036) and at 12 months of age (CD vs CD+LR, *P = *0.0036) (Fig. S2a). As expected, the absolute nuclear volume of Leydig cells per testis in those experimental groups was also significantly higher at 5 (CD vs CD+LR, *P = *0.0175; NWD vs NWD+LR, *P = *0.0012), 7 (CD vs CD+LR, *P = *0.0023; CD+*E.coli* vs CD+LR, *P = *0.0023) and 12 months of age (CD vs CD+LR, *P = *0.0026) (Fig. S2b). The relative percentages of nuclear volumes of Leydig cells per testis were significantly higher in the *L. reuteri*-fed mice at the age of 5 months (CD vs CD+LR, *P = *0.0111; NWD vs NWD+LR, *P = *0.0111), at the age of 7 months (CD vs CD+LR, *P = *0.0006; CD+*E.coli* vs CD+LR, *P = *0.0041) and at the age of 12 months (CD vs CD+LR, *P = *0.0126) (Fig. S2a). Therefore, the increase of the testicular Leydig cell population was disproportionally higher compared to the increase of testes weight in the mice consuming *L. reuteri*, which is highly suggestive of a particularly robust effect of the probiotic organism on Leydig cells.

In order to provide further evidence for this effect, we morphometrically examined the nuclear diameter of Leydig cells in the different experimental groups of mice. The size of Leydig cell nuclei was consistently higher in the groups of mice fed with *L. reuteri* at 5 months (CD vs CD+LR, P<0.0001; NWD vs NWD+LR, P<0.0001), at 7 months (CD vs CD+LR, P<0.0001; CD+E.coli vs CD+LR, P<0.0001) and at 12 months of age (CD vs CD+LR, P = 0.0009) (Fig. S2c).

By using the Floderus equation and the nuclear volumes of Leydig cells per unit size area, the Leydig cell number contained in size unit areas was calculated. Having determined the mean nuclear diameter of Leydig cells contained in testes of known volume (weight), we were next able to assess the mean nuclear volume and, finally, the total Leydig cell number contained in each testis. The analysis of these data showed that the number of Leydig cells per testis was higher in *L. reuteri-*treated mice at 5 months (CD vs CD+LR, P = 0.0963; NWD vs NWD+LR, P = 0.0262), at 7 months (CD vs CD+LR, P = 0.0041; CD+E.coli vs CD+LR, P = 0.0111) and at 12 months of age (CD vs CD+LR, P = 0.007) (Fig. 3c). Taken together, these results show that the more conspicuous Leydig cell areas characterizing the testes of *L. reuteri-*fed mice are due to increased numbers of Leydig cells which, in addition, bear nuclei of increased size.

**Figure 4:**
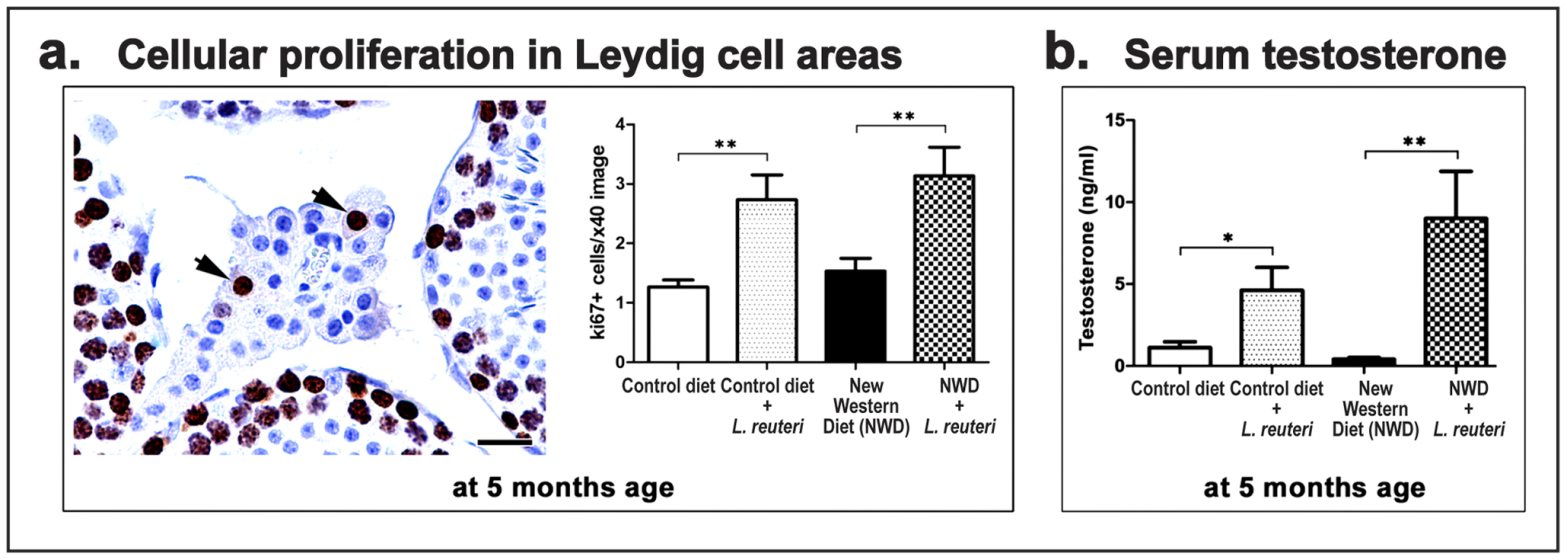
*L. reuteri* increases Leydig cell proliferation and serum testosterone levels. **a.** At the age of 5 months a small number of proliferating (Ki-67+) Leydig cells did exist in the testicular interstitium (arrows). Proliferating Leydig cells were significantly more in probiotic-fed mice. DAB chromogen, Hematoxylin counterstain. Bars = 25 µm. The y-axis of the bar graph stands for the mean±SEM of Ki-67+ Leydig cells. **b.** Serum testosterone levels are significantly higher when consuming *L. reuteri*, as compared to their diet-matched controls. Numbers on the y-axis corresponds to the mean±SEM of serum testosterone level in ng/ml.; *p<0.05, **p<0.001.

To further elaborate on this result we assessed immunohistochemically proliferation and apoptosis in the testes of 5 and 12 months-old mice. Apoptotic (Caspase3+) Leydig cells were practically undetectable at both time-points. Small numbers of proliferating (Ki-67+) cells, however, did exist in the testicular interstitium of 5 months-old mice. Ki-67+ cells had a large, round, centrally located nucleus and abundant vacuolated cytoplasm (Fig. 4a). Based on their typical morphology and the presumptive absence of foamy macrophages (which in granulation tissue might resemble vacuolated Leydig cells) these proliferating cells were identified as Leydig cells. Morphometric counts of ki-67+ Leydig cells in the different treatment groups revealed significant differences in proliferating Leydig cell counts of both control (CD vs CD+LR, *P = *0.0024) and NWD-fed groups of mice (NWD vs NWD+LR, *P = *0.0049) (Fig. 4a).

### *L. reuteri* Induces Elevation of Serum Testosterone

Given that the Leydig cell is the main cellular source of testosterone, we next examined the effect of the probiotic organism in testosterone serum levels of mice at the age of 5 months. We found that mice eating *L. reuteri* had profoundly increased levels of circulating testosterone regardless of the type of diet they consumed (CD mice vs CD+LR, *P* = 0.0266) and NWD-fed groups of mice (NWD vs NWD+LR, *P* = 0.0037) (Fig. 4b).

### *L. reuteri* Consumption Benefits Post-testicular Sperm Attributes

Testes histomorphometry and serum androgen concentration data were both suggestive of a probiotic-associated up-regulation of spermatogenesis in mice. To directly test this, we assessed sperm concentration and activity in sperm gathered form epididymites of mice at the age of 12 months. Sperm from *L. reuteri-*treated mice had significantly higher concentration (CD vs CD+LR, *P = *0.0286) and activity (CD vs CD+LR, *P = *0,0286) compared to non-treated age-matched control mice (Fig. S3).

### *L. reuteri* Counteracts Age-associated Testicular Atrophy

**Figure 5:**
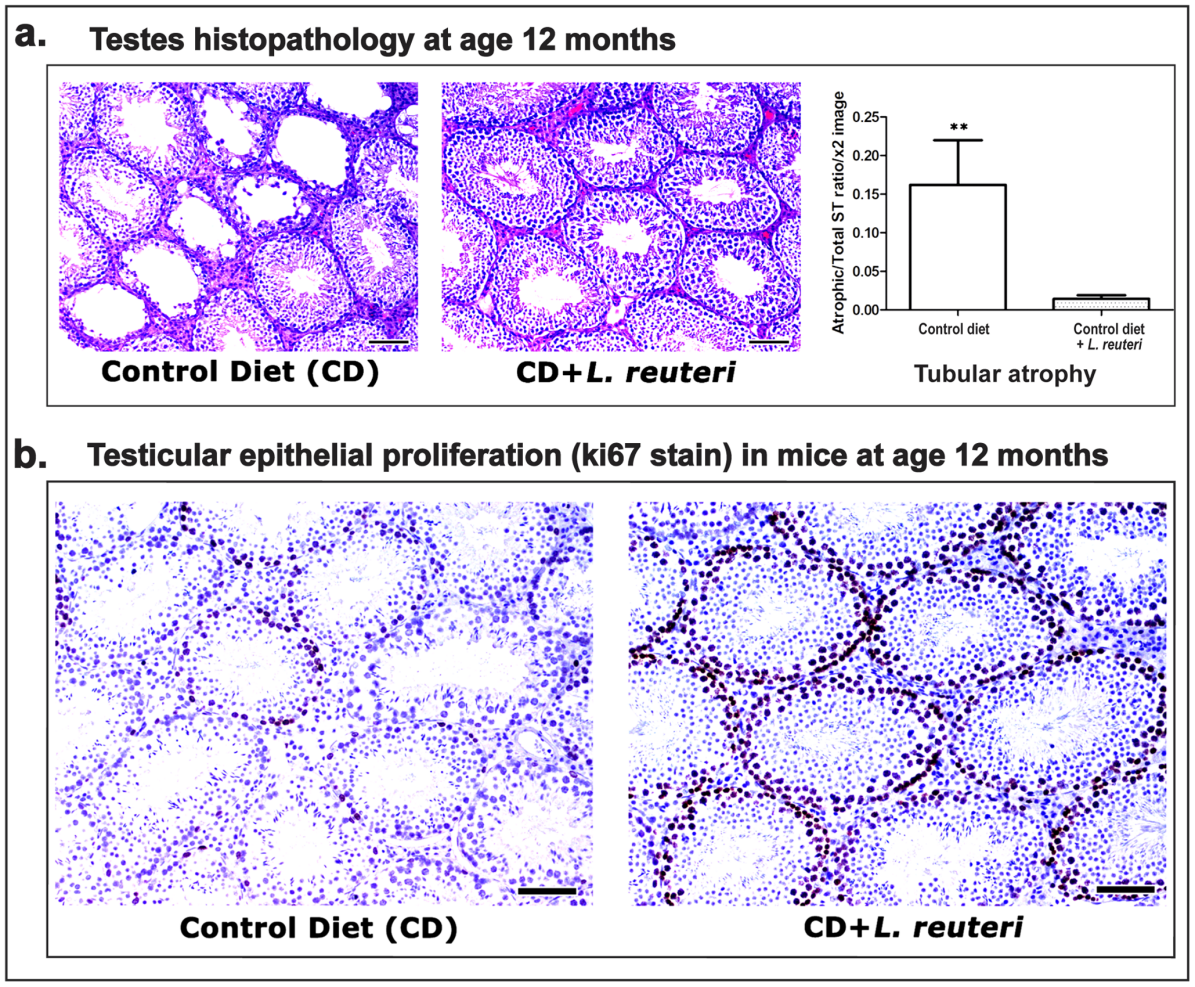
Dietary *L. reuteri* rescues mice from age-associated testicular atrophy. **a.** Representative histology of Swiss mouse testes at the age of 12 months. The aged control mouse testes demonstrated atrophic seminiferous tubules. By contrast, *L. reuteri*-fed mice had uniformly normal ST epithelium with rare atrophic tubules. Hematoxylin and eosin. Bars = 100 µm. The y-axis in the bar graph stands for the mean±SEM of the atrophic/total ST ratio counted in each experimental group;**p<0.001. **b.** Ki-67-specific immunohistochemistry in the testes of 12-months-old mice. A side-by-side comparison of representative images from control and *L. reuteri-*fed mouse testes reveals that the probiotic organism counteracts the age-associated decrease of epithelial proliferation observed in control mice. DAB chromogen, Hematoxylin counterstain. Bars = 100 µm.

Aged-related histopathological changes of spermatogenic epithelium have been described in both rodents and humans. In the present study, we found that mice eating control chow exhibited age-related changes in the testes at approximately the age of 7 months. These changes included ST epithelial vacuolation, segmental reduction of germ cells in ST profiles and the presence of small numbers of ST profiles lined only with Sertoli cells (end-stage atrophic tubules). At the age of 12 months the same lesions appeared with increased frequency and distribution (Fig. 5a). In addition, there were small number of tubules with atypical residual bodies and occasional ST's showing features of mineralization. In order to quantify the effect of *L. reuteri* in age-related atrophy of ST, we morphometrically measured the atrophic ST/Total ST ratio in the testes of mice at 12 months of age. We found that the atrophic tubule percentage was significantly less in mice fed with the probiotic organism compared to control mice (CD vs CD+LR, *P* = 0.0043) (Fig. 5a). Caspase-3-specific immunohistochemistry showed that germ cell apoptosis was unremarkable in both L. reuteri-treated and non-treated mice. Proliferating ki-67+ ST epithelial cells, however, were evidently increased in the mice consuming the probiotic organism (Fig. 5b).

### Blocking Il-17 Recapitulates the *L. reuteri* Effect on Mouse Testes

**Figure 6:**
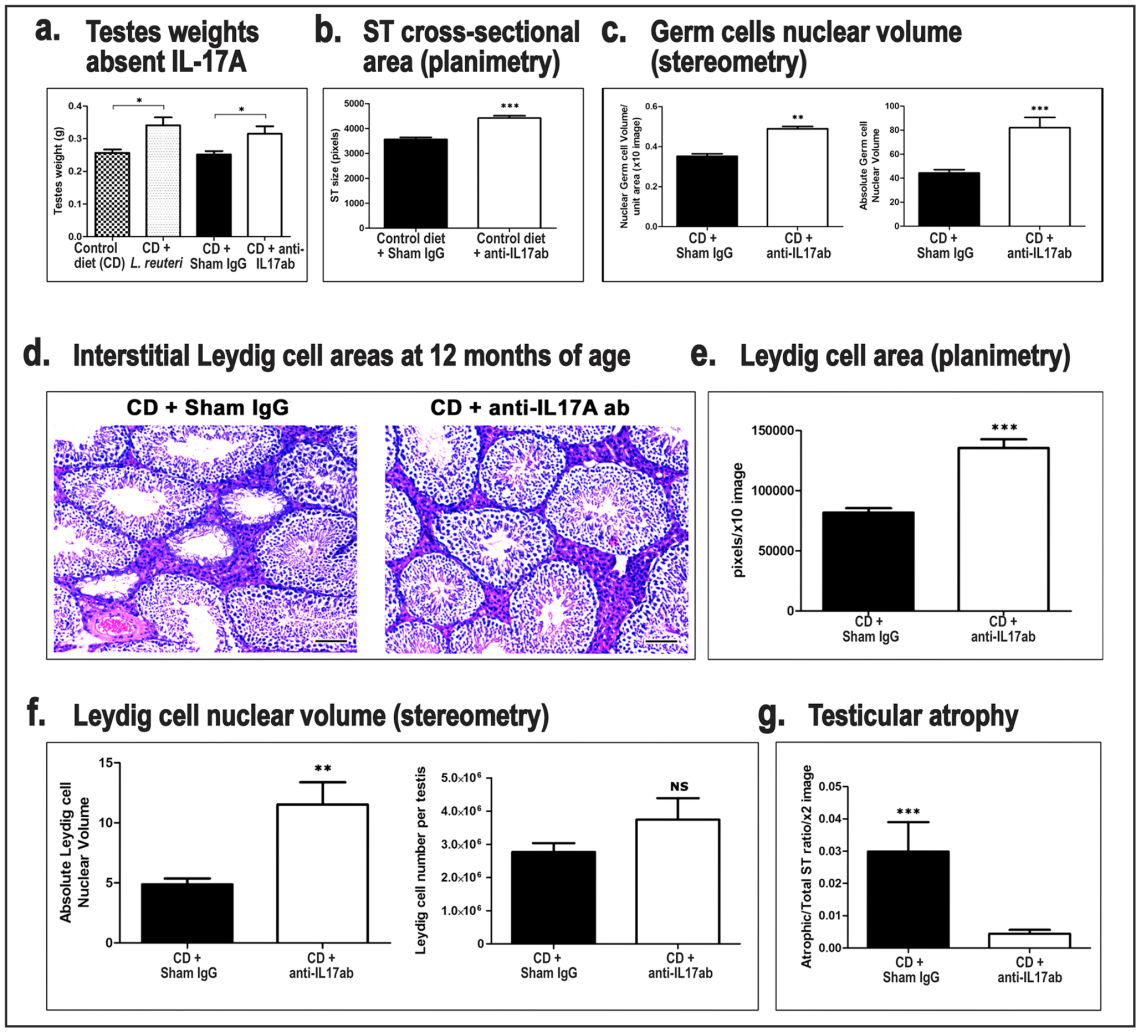
Systemic depletion of IL-17 recapitulates reproductive fitness attributes of *L. reuteri.* Compared to sham IgG-treated control mice, 12 month-old mice depleted of IL-17A had **a.** increased paired testicular weights; **b.** increased cross-sectional ST areas; **c.** increased germ cell nuclear volumes; **d and e.** increased interstitial Leydig cell areas. Hematoxylin and eosin. Bars = 100 µm; **f.** increased Leydig cell nuclear volumes and total number per testis and **g.** decreased ST atrophy. *p<0.05, **p<0.001, ***p<0.0001.

In our previous studies we found that dietary probiotics counteract obesity [19] and age-related integumentary pathology [18] at least in part by down-regulating systemic pro-inflammatory IL-17A-dependent signaling. To test whether beneficial effects of *L. reuteri* within testes also associate with this anti-inflammatory mechanism, we examined the effect of Il-17 depletion in the testes of 12-month-old mice. We found that blocking pro-inflammatory Il-17 signaling entirely recapitulates the beneficial effects of probiotics (Fig. 6). The weight of testes of Il-17-depleted mice was significantly higher than that of sham IgG-treated control mice (CD+anti-ShamIgG vs CD+anti-IL17, *P* = 0.0230) (Fig. 6a). The testis weight/body weight analysis had a similar outcomes (Fig. S1). As with feeding of L. reuteri, the anti-IL17 treatment had no effect in total ST number (data not shown), but it did affect significantly the cross-sectional profile size of ST (CD+anti-ShamIgG vs CD+anti-IL17, *P*<0.0001) (Fig. 6b). The point-counting stereometry method showed that nuclear volume of germ cells per unit size area (CD+anti-ShamIgG vs CD+anti-IL17, *P = *0.0021) and the absolute nuclear volume of germ cells per testis (CD+anti-ShamIgG vs CD+anti-IL17, *P = *0.0006) were also increased with IL-17 neutralization (Fig. 6c). As with probiotic dietary supplementation, IL-17 depletion did not affect the relative percentages of nuclear volumes of germ cells per testis (data not shown).

The Leydig cell area size also increased (Fig. 6d) with depleting mice from IL-17 (CD+anti-ShamIgG vs CD+anti-IL17, *P*<0.0001) (Fig. 6e) in a manner similar to *L. reuteri* treatment in age-matched Swiss male mice. This result was further confirmed by stereological Leydig cell nuclear volume measurements. According to these, the nuclear volume of Leydig cells per unit size area (CD+anti-ShamIgG vs CD+anti-IL17, *P = *0.003) (Fig. S2a), the absolute nuclear volume of Leydig cells per testis (CD+anti-ShamIgG vs CD+anti-IL17, *P = *0.0021) (Fig. 6f) and the relative percentages of nuclear volumes of Leydig cells per testis (CD+anti-ShamIgG vs CD+anti-IL17, *P = *0.0213) (Fig. S2b) were all significantly increased after depleting IL-17. Likewise, the nuclear diameter of Leydig cells was increased with this anti-inflammatory treatment (CD+anti-ShamIgG vs CD+anti-IL17, *P<*0.0001) (Fig. S2c). The total number of Leydig cells per testis was also increased. This difference, however, did not reach statistical significance (CD+anti-ShamIgG vs CD+anti-IL17, *P = *0.3706) (Fig. 6f).

Finally, the age-related atrophy exhibited per total ST percentage was ameliorated (CD+anti-ShamIgG vs CD+anti-IL17, *P*<0.0001) (Fig. 6g). These findings showed that systemic reduction in inflammation in the form of IL-17A was sufficient to mimic the testicular benefits of oral *L. reuteri* supplementation.

## Discussion

While conducting diet studies involving probiotic microbe Lactobacillus reuteri we discovered that aging male animals had larger testes compared to their age-matched controls [18]–[19]. This led us to hypothesize that dietary *L. reuteri* supplementation may act to prevent age- and diet-related testicular atrophy in mice. Knowing that age-related hypogonadism has been linked with functional alterations of Leydig cells and low testosterone levels that reduce reproductive fitness and quality of life [1], [4]–[5], [9]–[10], [12]–[13], we examined testes of probiotic microbe-fed mice and found that they had less testicular atrophy coinciding with higher levels of circulating testosterone compared to their age-matched controls. Similar testicular health benefits were produced using systemic depletion of the pro-inflammatory cytokine Il-17 alone, implicating a chronic inflammatory pathway in hypogonadism. Based on these observations, we propose a model whereby probiotic bacteria modulate gastrointestinal immunity resulting in systemic effects on the immune system that activate metabolic pathways that restore tissue homeostasis and overall health.

One specific aspect of this paradigm is reciprocal activities of pro-inflammatory Th-17 and anti-inflammatory Treg cells [21]. Along mucosal surfaces, anti-inflammatory cytokine Interleukin (IL)-10 facilitates immune tolerance [22] and recruitment of CD4+ Treg cells to skew host immunity away from pro-inflammatory IL-17. Feeding of *L. reuteri* organisms was previously shown to up-regulate IL-10 levels and reduce levels of IL-17 [19] serving to lower systemic inflammation. Conversely, insufficient levels of IL-10 may increase the risk for autoimmunity, obesity, and other inflammatory disease syndromes [23]–[27]. Indeed, many health disorders associated with Westernized life are believed due to insufficient IL-10 and improper host immune calibration resulting in uncontrollable inflammation [28]. Westernized diets are also low in vitamin D, a nutrient that when present normally works together with IL-10 to protect against inflammatory disorders [29]–[31] and even some types of cancer [32]. Physiological feedback loops apparently exist between microbes, host hormones, and immunity. The hormone testosterone has been shown to act directly through androgen receptors on CD4+ cells to increase IL-10 expression [33]. Leydig cells are the main cellular source of testosterone in male mammals that naturally decline during aging [2]–[4]. Although adult Leydig cell androgen production is regulated by LH-mediated signals originating from the hypothalamus-pituitary gland axis [1], studies in both humans and rodents suggest that hypogonadism is due to age-related lesions in testes rather than irregular LH metabolism [1], [5]–[7]. Further studies of LH and the hypothalamus-pituitary axis are underway to investigate this possibility.

We investigated spermatogenesis and other features associated with reproductive fitness [8]–[11], seeing significant retention of testicular heath criteria after feeding of *L. reuteri*. We postulate that probiotic gut microbes function symbiotically with their mammalian hosts to impart immune homeostasis to maintain systemic and testicular health [34]–[35] despite suboptimal dietary conditions. Dietary factors and diet-induced obesity were previously shown to increase risk for age-associated male hypogonadism, reduced spermatogenesis, and low testosterone production in both humans and mice [2]–[4], [8]–[11], [14]–[17], phenotypic features that in this study were inhibited by oral probiotic therapy absent milk sugars, extra protein, or vitamin D supplied in yogurt. Similar beneficial effects of probiotic microbes on testosterone levels and sperm indices were reported in male mice that had been simultaneously supplemented with selenium [36]. Although neither of these studies analyze testicles of human males, probiotic yogurt did produce leaner physiques in mice [19] matching findings of a large epidemiological survey of human subjects when eating yogurt [37] [38]–[43]. Additional studies using human subjects are needed to assess translational potential. A proposed health-protective role for *L. reuteri* in mammalian host metabolism offers mutually beneficial gut symbiont-host relationship reflecting co-evolutionary relationships [44].

From an evolutionary perspective, we assert that lactic acid bacteria may have co-evolved with mammals exploiting testosterone to optimize mental, physical, and reproductive fitness. Higher serum testosterone levels compared to controls in our separate studies correlated with not only leaner physique but also increased muscle mass and higher activity levels in mice (data not shown). Benefits of this microbial synergy may extend beyond individual fitness to reproductive success, impacting a natural selection process favoring evolutionary success for the microbe and mammalian host.

Our data suggest that the *L. reuteri-*associated prevention of age- and diet-related testicular atrophy correlates with increased numbers and size of Leydig cells. At the earliest time-point examined in the present study (age = 5months), we found no histological evidence of age-related testicular atrophy in mice. This matches the results of a previous study, which describes that the initial changes of testicular atrophy begin to occur in mice from the age of 6 moths onwards [7] and indicates that the trophic effect of *L. reuteri* on Leydig cells is a key event which precedes and prevents age-related changes in the testes of mice. This effect is reminiscent of earlier studies describing Leydig cell hyperplasia and/or hypertrophy in the mouse and the rat testis that were achievable by the administration of gonadotropins, including human chorionic gonadotropin, FSH and LH [45]–[49].

The similarity of the effects of hypopheseal hormones and *L. reuteri* administration on Leydig cells warrant further investigation, especially in the light of recent evidence for the effects of edible Lactobaccili in the hypothalamus and pituitary gland [50]–[51]. In human males as well as in Brown Norway rats, a strain that is often used to study the effects of aging in Leydig cells, the age-associated decrease of serum testosterone does not co-exist with decreased plasma luteinizing hormone (LH) concentrations. This indicates that sub-normal testosterone levels are due to testicular dysfunction rather than a disruption of the hypothalamus-pituitary axis. The primary gonadal deficit was found to result from compromised Leydig cell steroidogenesis, but not from their loss [1], [4]–[6]. Studies in other strains of rats as well as recent studies in humans, however, suggest that LH and the hypothalamic-pituitary level are important in male hypogonadism [1], [4]–[6], [52]. These discrepancies make it difficult to determine whether the *L. reuteri*-induced effects found in mice in the present study could be directly relevant to aging human males. The restoration of testosterone levels, if proven true as an *L. reuteri*-induced effect in aged humans, would directly benefit their health regardless to whether the level of the *L. reuteri* intervention in the hypothalamus-pituitary-testis axis [9], [11], [16], [20].

Taken together, our data indicate that probiotic organisms may offer practical options for management of disorders frequently associated with normal aging. Reduced circulating testosterone levels have been implicated in many adverse effects including reduced spermatogenesis, libido and sexual function, increased body fat, decreased muscle and bone mass, low energy levels, fatigue, poor physical performance, depressed mood, and impaired cognitive dysfunction [8]–[11]. Ultimately, dietary *L. reuteri* or other probiotic supplementation may provide an alternative natural approach to prevention of male hypogonadism, absent the controversy and side-effect risks of testosterone replacement therapy [9]–[10], [13], [20]. Such microbial immune system re-programming may ultimately target other diseases linked to low testosterone including increased body fat, decreased muscle and bone mass, weak physical and mental performance, and depressed mood [8]–[11] for more healthful longevity.

## Experimental Procedures

### Animals

Genetically outbred CD-1 mice (Charles River; Wilmington, MA) were housed and handled in Association for Assessment and Accreditation of Laboratory Animal Care (AAALAC)-accredited facilities with diets, experimental methods, and housing as specifically approved by the Institutional Animal Care and Use Committee. The MIT CAC (IACUC) specifically approved the studies as well as the housing and handling of these animals. Mice were bred in-house or purchased directly from the vendor to achieve experimental groups. Mice were housed in groups of four animals per cage, except for those animals specifically used in breeding experiments.

The experimental design was to expose mice to diets starting at age = eight weeks, and then continue the treatment until euthanasia using carbon dioxide: 1) for 12 weeks to achieve the cohort collected at five months of age; 2) for 20 weeks to achieve the cohort collected at seven months of age; 3) for 28 weeks to achieve the cohort collected at nine months of age; and 4) for 40 weeks to achieve the cohort collected at 12 months of age. Each experiment included 5–10 animals per group with at least one replicate for each experiment. Necropsy procedures were performed in mid-afternoon to standardize collections relative to circadian rhythms. After humane euthanasia with CO_2_ overdose, 1 ml of blood was collected using cardiac puncture method in unconscious animals. Body weights of whole mice or their isolated paired testes were recorded (Scout pro sp202; Ohaus Corporation, Pine Brook NJ) for comparisons between treatment groups.

### Special Diets for Animals

Mice of 6–8 wks were placed on experimental diets: control AIN-76A (Harlan-Teklad Madison WI), and a Westernized diet with high fat and low fiber with substandard levels of Vitamin D (TD.96096; Harlan-Teklad) starting at 8 weeks of age until euthanasia at time points of five months of age, seven months of age, nine months of age, or 12 months of age. Separate groups of animals received a purified preparation of an anti-inflammatory strain of *Lactobacillus reuteri* ATCC PTA 6475 cultivated as described elsewhere [53] using a dosage of 3.5×10^5^ organisms/mouse/day, or a sham *E coli* K12 3.5×10^5^ organisms/mouse/day in drinking water. Drinking water was replaced twice weekly.

### Systemic Depletion of Interleukin-17A using Anti-cytokine Antibodies

Mice at age 6 months or older were treated with intraperitoneal injection of anti-IL17A antibody (clone 17F3; BioXcel, Lebanon, NH) at 500 µg per mouse three times weekly for at least four weeks. Mice were then euthanized and compared with age-matched mice that received isotype-matched sham IgG antibody alone.

### Measurements of Serum Testosterone

Terminal cardiac blood collections were performed upon euthanasia in mid-afternoon to normalize for Circadian rhythm effect on testosterone. RadioImmunoAssay (RIA) was performed using Coat-A-Count Total Testosterone RIA kit (Siemens Diagnostics CA) as performed by AniLytics Inc (Gaithersburg MD).

### Quantification of Sperm Quality

After humane euthanasia using C0_2_, both testes were isolated to remove combined epididymal and vas tissues from a particular male mouse. Tissue was immediately placed into a plastic Petri dish and immersed in 1 ml FHM solution (Specialty medium, Millipore). Under dissecting microscope, sperm were mobilized from each epididymis and vas deferens using microdissection with a 16 G needle. The dish was placed to a CO_2_ incubator at 37°C for 10 minutes to allow for complete sperm mobilization. Sperm were then suspended 1∶10 with FHM to evaluate sperm concentration using a hemocytometer (Fisher). Sperm concentration/ml was calculated using (Dilution factor)×(count number in 5 squares)×(0.05×10^6^). Sperm activity was assessed as number of actively moving sperm/total number of sperm [54]–[56].

### Histopathology and Immunohistochemistry

For histologic evaluation, testes were fixed in neutral-buffered formalin, cut transversely in half, embedded in paraffin and cut at 4 µm or at 10 µm for stereology. Serial sections were stained with hematoxylin and eosin and immunohistochemistry (IHC). Rabbit monoclonal anti-Ki-67 (Cell Marque, Rocklin, CA) or polyclonal anti-cleaved caspase-3 (Cell Signaling, Beverly, MA) antibodies were used for IHC. Heat-induced antigen retrieval was performed with CC1 epitope retrieval solution (Ventana Medical Systems, Inc., Tucson, AZ) for ki-67 or with citrate buffer pH6 for caspase-3.

Quantitative histomorphometry was done using planimetry-based morphometry for the assessment of seminiferous tubules (ST) cross-sectional areas, the Leydig cell areas and nuclear diameters and for total ST profiles and atrophic/total ST profile measurements. A stereology-based point-counting morphometry method [57] was applied for determining germ and Leydig cell nuclear volumes. Six to eight histological sections from each mouse testes were randomly selected after step-sectioning and used for the morphometric counts. Images taken at ×2 magnification were used for ST cross-sectional areas and for total ST and atrophic/total ST measurements. ×10 magnification images were used to measure Leydig area size and for germ and Leydig cell nuclear volume stereology, ×40 for ki67+ Leydig cell counts and ×60 for determining Leydig cell nuclear diameters. Images were analyzed using the Image J image processing and analysis program (NIH, Bethesda, MD). For ST cross-sectional areas, for total ST and atrophic/total ST and for ki67+ Leydig cell counts, depending on the experimental group size, 1–3 images were randomly selected from each mouse to achieve a total number of 25 images per group. Total ST profiles, atrophic ST profiles and ki-67+ Leydig cells were counted in each image. For ST cross-sectional area planimetry, the largest 4–5 perfectly circular in shape ST profiles found in each image were subscribed and each ones' area was automatically measured in pixels using the ImageJ “measure” command. A total number of at least 100 ST cross-section area values was achieved per group. For Leydig cell area planimetry, the 3 images containing the largest proportion of Leydig cell areas from each mouse were used. Depending on the group size 1–3 images were randomly selected from each mouse to achieve a total number of 25 images to work with per group. The multiple Leydig cell areas in each image were subscribed, excluding vessels or “empty” spaces, and measured. The total Leydig cell area per image was recorded. For Leydig cell nuclear diameter measurements, 5 images from randomly selected areas of each mouse testis were captured using the ×60 high power magnification lens. Depending on the experimental group size, the diameter of 10–20 circular Leydig cell nuclear profiles were measured to achieve 200 nuclear size values per treatment group. For germ and Leydig cell point-counting, seven mice were randomly selected from each experimental group. Ten ×10 images were randomly captured from each mouse testis. Specifically, from each one of the 6–8 histological sections available for that assessment, the first 1 to 2 fields found to contain target cells after a random movement of the microscope stage with a closed field diaphragm were captured (simple random sampling)”. A grid with 130 points was super-imposed in each image using the “grid” plug-in of the ImageJ software. The number of points co-localizing with germ or Leydig cell nuclei was counted in each image. The mean count of nuclei-positive points found in the 10 images was determined for each mouse. The ratio of the mean “nuclei-positive points”/130, determined the “Nuclear volume of cells per unit area” (×10 figure) for a given mouse. The product of “Nuclear volume per unit area” of a mouse testis multiplied by the testis weight (“fresh” testis weight) determined the “absolute nuclear volume of cells” in each mouse testis. The quotient “Nuclear volume of cells per unit area”/Testis weight expressed as percentage was the “Relative percentage of nuclear volume of cells” of each testis. In order to calculate the total number of Leydig cells per testis, the mean nuclear diameter of 100 randomly selected circular profiles of Leydig cells was determined for each testis of the mice used for stereometry (n = 7 per group). The mean diameter in pixels was converted to µm and the weight ( = volume) of each testis was expressed in µm^3^. Then the Floderus equation N_V_ = N_A_/(T+D−2 h) was used to transform the “Nuclear volume of cells per unit area” (N_A_). T is the section thickness, D is the mean Leydig cell diameter and h is the height of the smallest nuclear profile found in the section. The latter was arbitrarily set at the 0.1× mean nuclear diameter [58]. The total number of Leydig cells per unit area (N_V_) of each testis was multiplied by the testis volume (assuming that each testis volume equals to its weight) to determine the total number of Leydig cells per testis.

#### Statistical analyses

The Mann-Whitney U test was used for body and testes weights and histomorphometry. A p-value <0.05 was statistically significant.

## Supporting Information

Figure S1 ***L. reuteri*** **increases testicular weight/body weight ratio.** Dietary supplementation with *L. reuteri*, increased testicular weight-body weight ratio when compared to age- and diet-matched controls. A similar effect was observed with the neutralization of IL17. Numbers on the y-axis of bar graphs correspond to the mean±SEM of the testicular weight/body weight ratio. *p<0.05, **p<0.001, ***p<0.0001.
(TIF)

Figure S2 **Effects of** ***L. reuteri*** **on Leydig cells. a.** *L. reuteri*-fed mouse testes and mice undergoing depletion of IL-17 have a significantly increased Leydig cell nuclear volume per size unit area and **b.** increased relative percentage of Leydig cell nuclear volume compared to control mice. Numbers on the y axis of bar graphs correspond to the mean±SEM of “relative percentage of Leydig cell nuclear volume”;. **c.** In a similar manner, the same mice show an increased Leydig cell nuclear diameter compare to their age-, diet- and treatment-matched controls. Numbers on the y axis of bar graphs correspond to the mean±SEM of Leydig cell nuclear diameter. *p<0.05, **p<0.001, ***p<0.0001
(TIF)

Figure S3 **Dietary** ***L. reuteri*** **increases spermatogenesis in mice.** *L. reuteri* has a beneficial effect in sperm concentration and sperm activity of 12-month-old outbred Swiss mice. *p<0.05, **p<0.001.
(TIF)
