# Supplementary figures and images for: Probiotic Microbes Sustain Youthful Serum Testosterone Levels and Testicular Size in Aging Mice
Source: PLoS One. 2014 Jan 2;9(1):e84877. doi: 10.1371/journal.pone.0084877 (PMC3879365; doi:10.1371/journal.pone.0084877)

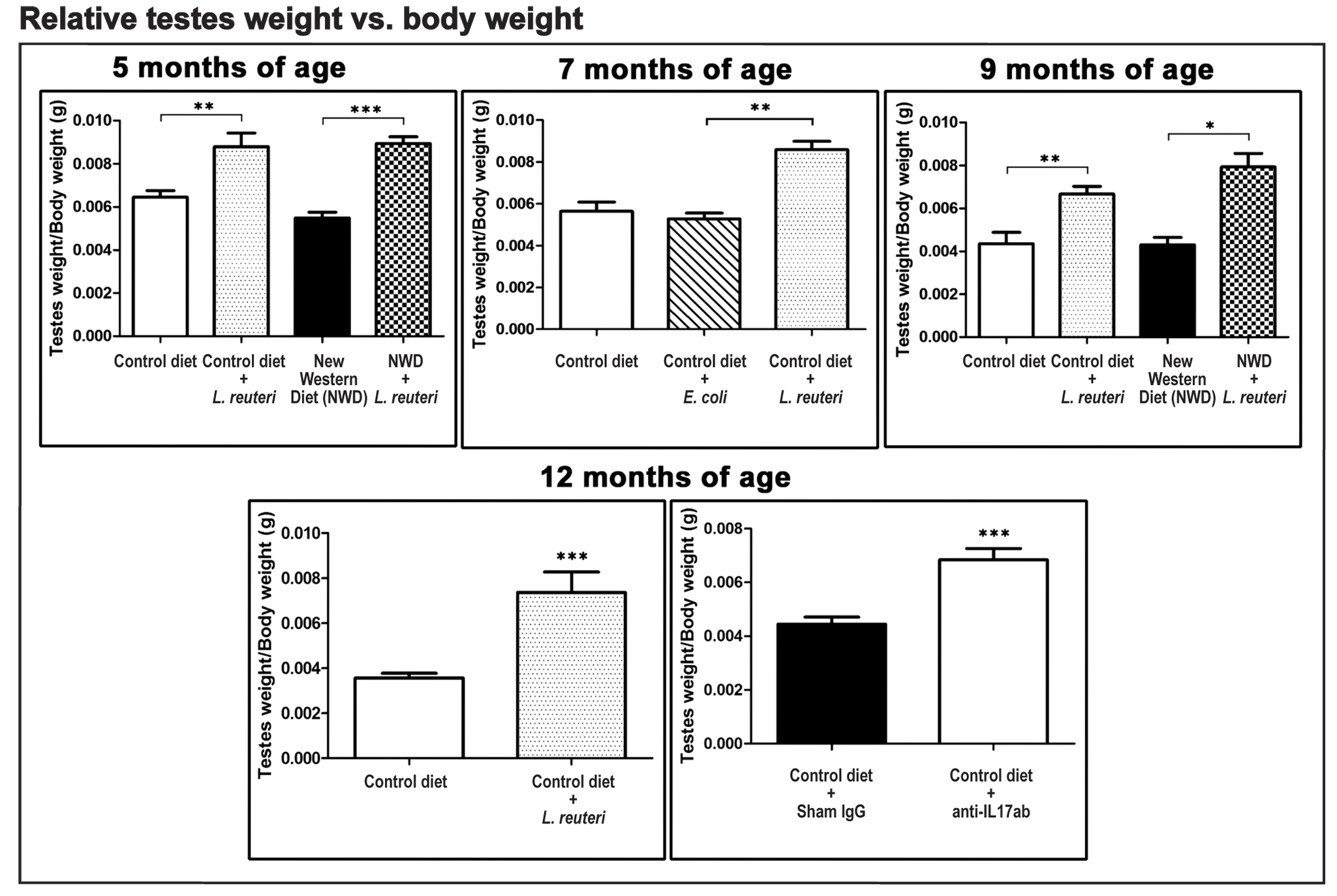

Supplement: Figure S1 — L. reuteri increases testicular weight/body weight ratio. Dietary supplementation with L. reuteri, increased testicular weight-body weight ratio when compared to age- and diet-matched controls. A similar effect was observed with the neutralization of IL17. Numbers on the y-axis of bar graphs correspond to the mean±SEM of the testicular weight/body weight ratio. *p<0.05, **p<0.001, ***p<0.0001. (TIF) [file pone.0084877.s001.tif]

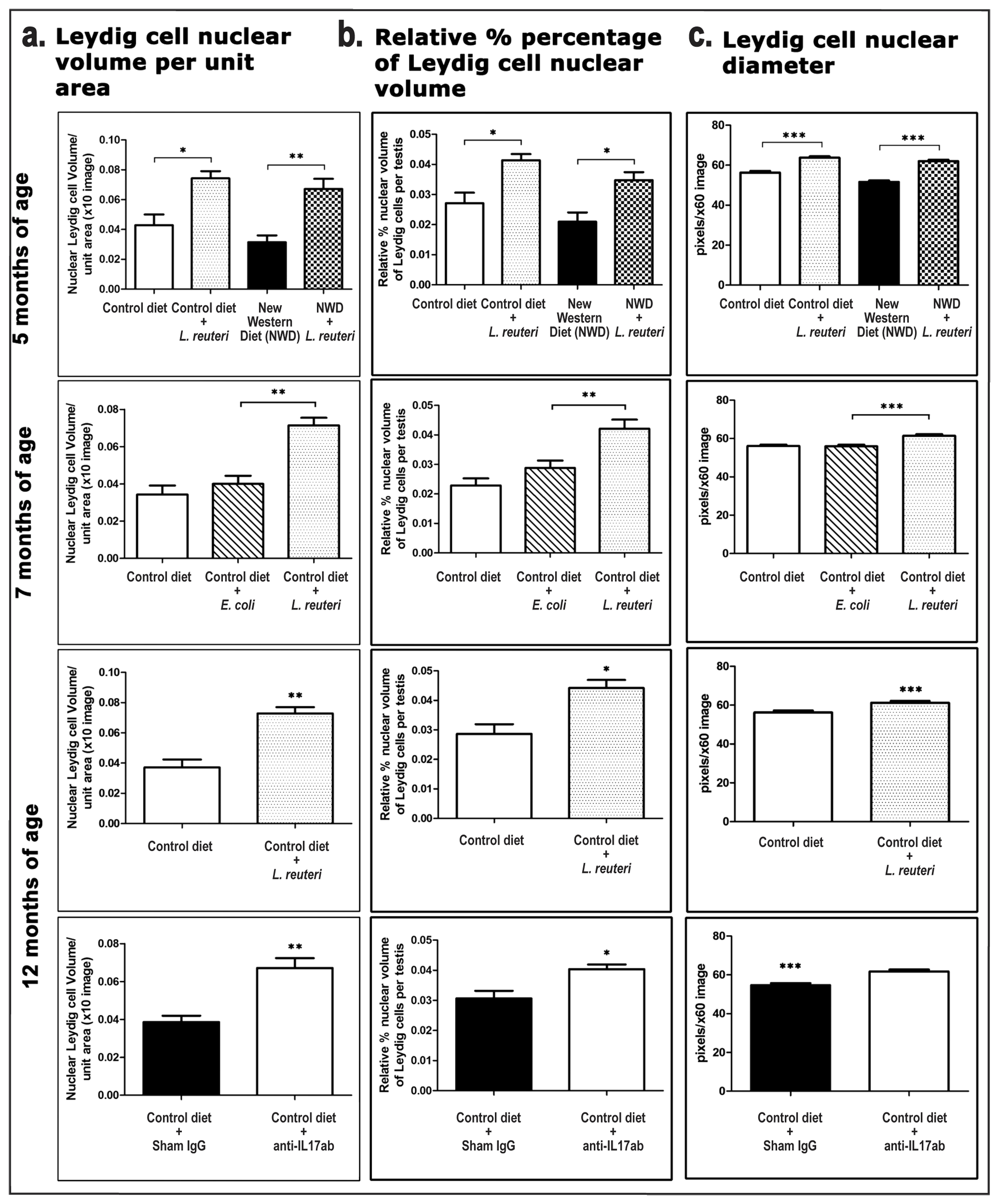

Supplement: Figure S2 — Effects of L. reuteri on Leydig cells. a. L. reuteri-fed mouse testes and mice undergoing depletion of IL-17 have a significantly increased Leydig cell nuclear volume per size unit area and b. increased relative percentage of Leydig cell nuclear volume compared to control mice. Numbers on the y axis of bar graphs correspond to the mean±SEM of “relative percentage of Leydig cell nuclear volume”;. c. In a similar manner, the same mice show an increased Leydig cell nuclear diameter compare to their age-, diet- and treatment-matched controls. Numbers on the y axis of bar graphs correspond to the mean±SEM of Leydig cell nuclear diameter. *p<0.05, **p<0.001, ***p<0.0001 (TIF) [file pone.0084877.s002.tif]

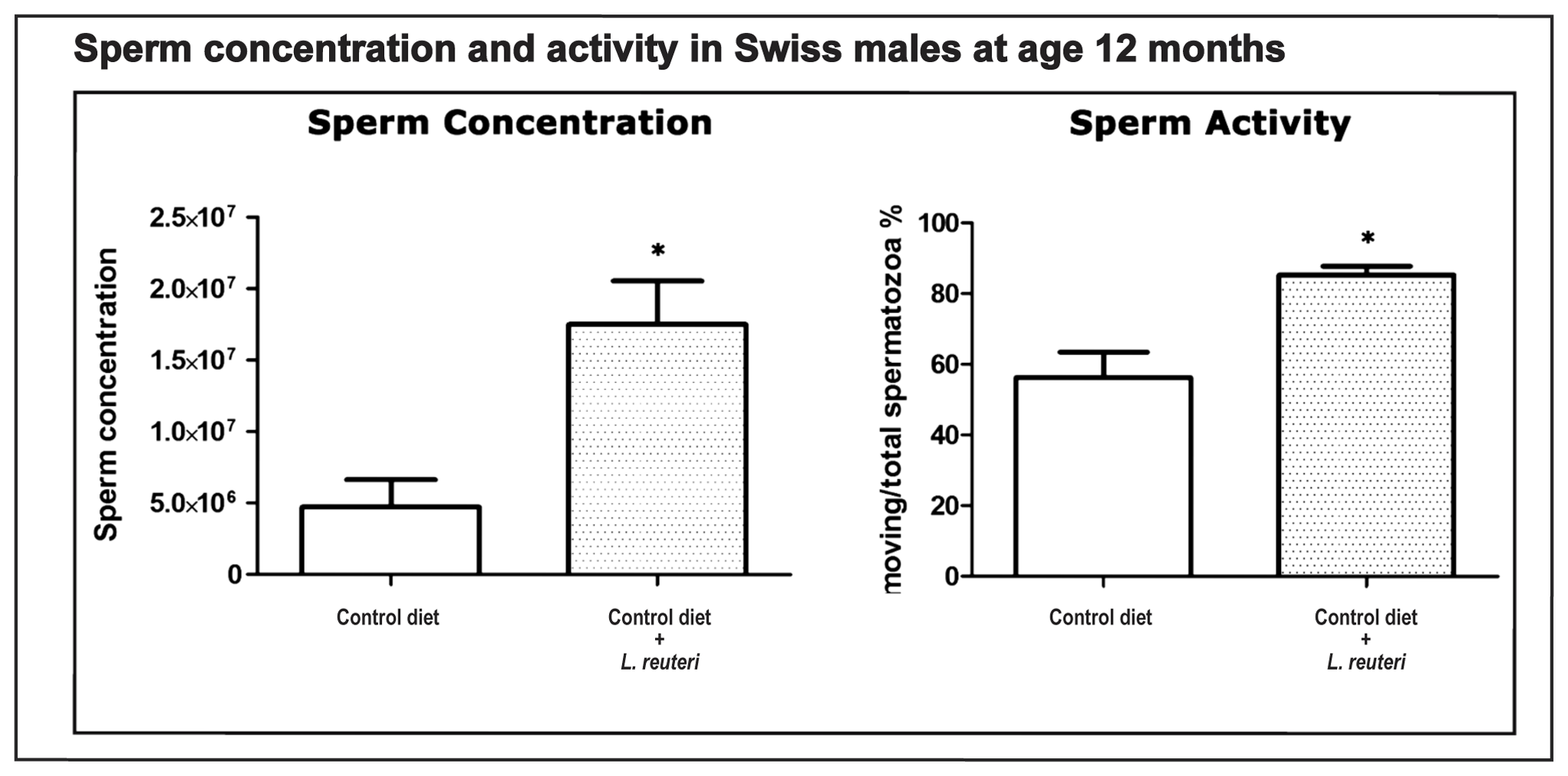

Supplement: Figure S3 — Dietary L. reuteri increases spermatogenesis in mice. L. reuteri has a beneficial effect in sperm concentration and sperm activity of 12-month-old outbred Swiss mice. *p<0.05, **p<0.001. (TIF) [file pone.0084877.s003.tif]
